# Genomic Characterization of Two Bovine Enterovirus Strains Isolated from Newly Transported Cattle

**DOI:** 10.3390/vetsci12070660

**Published:** 2025-07-11

**Authors:** Cuilan Wu, Shuhong Zhong, Shiwen Feng, Huili He, Shuai Hu, Zhongwei Chen, Changting Li, Xiongbiao Xuan, Hao Peng, Zuzhang Wei, Jun Li

**Affiliations:** 1 Institute of Animal Science and Technology, Guangxi University, Nanning 530005, China; cuilanwu@163.com; 2 Guangxi Key Laboratory of Veterinary Biotechnology, Guangxi Veterinary Research Institute, Nanning 530001, China; zhongshuhong11@163.com (S.Z.); fengshw1205@163.com (S.F.); hhl19870820@163.com (H.H.); benlaolao0000@163.com (S.H.); chen_zhong-wei@163.com (Z.C.); lctyq0508@163.com (C.L.); xuanxiongbiao@163.com (X.X.); 3 Key Laboratory of China (Guangxi)-ASEAN Cross-Border Animal Disease Prevention and Control, Ministry of Agriculture and Rural Affairs of China, Nanning 530001, China

**Keywords:** bovine enterovirus, genomic characterization, phylogenetic analysis, physical and chemical properties, newly transported cattle

## Abstract

This study isolated and identified two novel Chinese bovine enterovirus (BEV) strains, designated as BEV-GX1901 and BEV-GX1902, from newly transported cattle with the diarrheal feces symptom. We also determined their complete genome sequences (7408 and 7405 nucleotides, respectively) and found both strains have a genome organization analogous to that of picornaviruses. To better understand these two novel strains, a detailed analysis was applied to both strains, including the time of the cytopathic effect (CPE) production, TCID_50_ measurement, trypsin sensitivity test, ether sensitivity test, chioroform sensitivity test, acid and alkali resistance test, and heat resistance test. Our results showed that these two strains are different in physical and chemical properties. Our study also characterized that BEV-GX1901 and BEV-GX1902, both belonging to the BEV-E4 subtype, were closely related to the Australian strains K2577 and SL305, and the Japanese strain IS1 based on their genome sequences and VP1 region characterizations. It is speculated that this may be related to cattle trade and transportation. Additionally, the gene-by-gene or amino acid-by-amino acid comparison of the two strains found they have differences between their 5′UTR, 3′UTR, VP2, VP1, 2A, 3C, and 3D regions. Our results provide an important update of the virus’s presence in China and contribute to a better understanding of the distribution and characterization of BEVs in cattle.

## Simple Summary

This study addresses the lack of genomic data on bovine enteroviruses (BEVs) in transported cattle, which pose potential risks for disease transmission in livestock trade. We isolated two EV-E4 strains (BEV-GX1901 and BEV-GX1902) from diarrheal feces of newly transported cattle in China, aiming to characterize their genomic features, physicochemical properties, and phylogenetic relationships. The complete genomes (7408 nt and 7405 nt, respectively) revealed a typical picornavirus organization, showing different physical and chemical properties. The phylogenetic analysis showed that two strains were similar to EV-E4 strains (Australian strains K2577, SL305, and Japanese strain IS1), sharing a 76.7–90.9% VP1 nucleotide identity, with notable genomic variations in non-coding regions and structural proteins. These findings represent the first report of EV-E4 in transported cattle in China, suggesting possible links to the international livestock trade. Our results provide important updates on the prevalence of this virus in China and enhance the understanding of the BEV distribution and characteristics in cattle populations. In addition, this study also provides a scientific basis for disease control policies in the global livestock trade.

## 1. Introduction

Bovine enteroviruses (BEVs), which belong to the *genus* Enterovirus within the family Picornaviridae, are small, non-enveloped viruses that consist of a single-stranded positive-sense RNA genome and the surrounding capsid proteins. The genome has approximately 7500 nucleotides (nt), which contains a single open reading frame (ORF) flanked by untranslated regions (UTRs) at both the 5′ and 3′ ends. The ORF encodes a single long polyprotein that contains both structural proteins (VP1-VP4 encoded in P1) and non-structural proteins (2A, 2B, and 2C encoded in P2 as well as 3A, 3B, 3C, and 3D encoded in P3) [1].

The *genus* Enterovirus can be divided into 15 species, including the Enterovirus A-L (EV-A, B, C, D, E, F, G, H, I, J, K, and L) and Rhinovirus A-C (RV-A, B, and C) [2]. BEVs belong to the EV-E and EV-F families (formerly known as BEV-A and BEV-B, respectively) [3]. EV-E and EV-F can be further divided into several subtypes based on their genotype characterizations: EV-E comprises five recognized subtypes (E1–E5), while EV-F contains seven recognized subtypes (F1–F7) [3,4,5].

The BEV was originally isolated by Moll & Davis [6]. Since then, BEVs have been reported in various regions globally, including Australia, Germany, Spain, the United Kingdom, and the United States [2,5,6,7,8,9,10,11]. BEVs have been predominantly isolated from cattle feces and also from the feces of other animals such as sheep, goats, horses, geese, possums, and deer. Despite being frequently isolated from a wide range of animal hosts across different geographical regions, the pathogenicity and virulence of EV-E and EV-F in animal hosts remain controversial as they are not only isolated from apparently healthy animals but also in animals with clinical symptoms including diarrhea, pyrexia, dehydration, and weight loss. Thus, the pathogenicity and virulence of BEVs in cattle diarrhea still need to be further investigated, particularly because this disease can lead to significant economic losses in the cattle industry, decreasing milk production and leading to the weight loss of animals. Some previous studies failed to experimentally reproduce the bovine enterovirus infection in calves nor reproduce obvious clinical symptoms, therefore, jumping to the conclusion that BEV was not a significant pathogenic agent for cattle. However, with an increasing number of BEV strains being isolated and identified from either the fatal enteric or respiratory diseases, the pathogenicity and virulence of BEV relating to these illnesses have been intensively explored recently [7,12,13,14,15].

Since BEV-E can be an important pathogen that causes calve diarrhea, studying the biological characteristics of the new virus provides theoretical and practical support for understanding the virus immune escape, transmission capacity, pathogenicity, etc. It also provides scientific basis data for a nucleic acid antigen reagent evaluation, vaccine development, and epidemic prevention and control. In this study, we isolated two novel BEV strains from fecal samples obtained from newly transported cattle with severe diarrhea. Madin–Darby bovine kidney (MDBK) cells were used to isolate the virus. After we screened for the presence of BEV using a reverse transcription (RT)-PCR, complete genome sequences were determined and phylogenetic trees were constructed to characterize the genomic features of the virus. In addition, pairwise identities of two BEVs were also analyzed.

This is the first time that two BEV strains have been successfully isolated from newly transported cattle. This will enhance the understanding of the characterization and genetics of BEV. It will enhance the understanding of the infection, prevalence, and virulence of BEV.

## 2. Materials and Methods

### 2.1. Collection of Samples and Isolation of Viruses

Fecal samples were collected from one farm originally introduced from Australia with diarrhea symptoms only. Potential infection with bovine coronavirus (BCV), bovine astrovirus (BoAstV), bovine kobuvirus (BKV), bovine rotavirus (BRV), or bovine viral diarrhea virus (BVDV) was also suspected. One gram of feces per sample was diluted in 9 mL PBS to prepare a 10% fecal suspension and centrifuged at 10,000× *g* for 10 min to collect the supernatant. The supernatant was then filtered (0.22 μm) while the flow-through was used to inoculate MDBK cells. The inoculum was discarded after incubation with MDBK cells for 1 h, and the cells were raised with Hank’s solution before adding Dulbecco’s modified eagle’s medium (DMEM) (Invitrogen, Carlsbad, CA, USA), which was supplemented with 10% fetal bovine serum (GIBCO), 2 mg/mL gentamycin, and 2 mM L-glutamine (Invitrogen). Inoculated cells were observed for the appearance of cytopathic effects (CPEs) daily under a light microscope. The virus was collected and passaged after the development of 80% cytopathic effects.

### 2.2. Purification of Viruses

The stock solution of viruses was diluted by 10 times and aliquoted into 0.1 mL each. One aliquot was inoculated onto MDBK cell culture in monolayer. After incubation for 1 h 37 °C, the monolayer culture of MDBK cells was covered with DMEM containing 2% agar. These cells were then incubated with the virus for 2–4 days until a significant cytopathic effect could be visualized. Then, three clear and low-density plaques were selected and cultured in serum-free DMEM. After repeated freezing–thawing 3 times, plaques were inoculated into MDBK cell monolayers separately. After three passages, the purified virus was obtained.

### 2.3. RNA Purification, cDNA Synthesis, and PCR Amplification

All fecal samples and the infected MDBK cells after purifying by plaque assay above were lysed in TRIzol reagent (Cowin, Beijing, China). The total RNA of each sample was extracted using a viral RNA extraction kit (OMEGA, Norcross, GA, USA). RT-PCR was performed using M-MLV 1st strand cDNA Synthesis Kit (TaKaRa, Shiga, Japan) following the manufacturer’s instructions. The cDNA samples were tested for BCV, BoAstV, BKV, BRV, BVDV, and BEV by PCR using specific primers.

For each of those samples with positive results, we did a complete genome amplification using the long-range RT-PCR method combined with the primer walking technique, and we gained two BEVs, which we named as BEV-GX1901 and BEV-GX1902.

### 2.4. Infection, Physical, and Chemical Properties of the Virus

To further identify their infection efficacy, each viral strain was diluted into a 10^6^ serial dilution to infect cells cultured in 96-well plates. For each viral strain, quadruplicates of wells were applied for each dilution rate. Following that, cells were cultured for 48 h. After that, the time of cytopathic effects (CPEs) was observed and counted, and the TCID_50_ was calculated following the standard procedure. The infection, physical, and chemical properties were determined by comparing the TCID_50_ (6). The sensitivity to 0.25% trypsin was assayed following the treatment of 1 mL virus suspensions with an equal volume of 0.5% trypsin for 1 h at 37 °C. Two viruses were treated either with organic solvents (chloroform and ether) and heated at 50 °C and 56 °C for 1 h or incubated at pH 3.0, pH 5.0, pH 9.0, and 10.0 before infecting the cells. The response to trypsin, heat, acid, or organic solvents was determined by comparing the TCID_50_ for treated and control groups.

### 2.5. Alignment and Phylogenetic Analysis

The whole genomes of BEV-GX1901 and BEV-GX1902 strains were sequenced, respectively. The whole genome data and the VP1 nucleotide sequences were compared with sequences of other BEV reference strains (NIH-GenBank). Nucleotide sequences of both strains were compared online using the ClustalW 2.1 program. Phylogenetic analysis among different BEVs was performed by using the neighbor-joining method in MEGA7 software.

## 3. Results

### 3.1. Virus Isolation and Purification

After incubating for 24 h, MDBK cells demonstrated round shrinkage and exfoliation. And 90% of the cells were exfoliated on the third day after incubation, whereas control cells were normal without lesions. To rule out the possibility of a toxin effect from the sample, the cultures with inoculum were blindly passaged for four generations, and a similar cytopathic effect was observed for each passage, indicating that the CPE was the result of a pathogen in the inoculum. After three rounds of purification, two strains of the virus were obtained.

### 3.2. RT-PCR Amplification

The RT-PCR of infected cell culture fluids and fecal samples indicated the absence of BCV, BoAstV, BKV, BRV, or BVDV, but only the BEV was detected by this test.

To further characterize the viruses, the complete nucleotide sequences of BEV isolates were determined using several pair primers. After sequencing and assembling the PCR-amplified overlapping fragments, the complete genome sequences of BEV-GX1901 and BEV-GX1902 both showed a typical picornavirus genome organization.

The complete genome length of BEV-GX1901 was 7408 nt, excluding the poly(A) tail. A large ORF of 6525 nt, which encoded a 2175 aa long polyprotein precursor, was flanked by an 814 nt long 5′UTR and a 69 nt long 3′UTR. The P1, P2, and P3 regions were 2517 nt (839 aa), 1734 nt (578 aa), and 2265 nt (755 aa) long, respectively. The base composition of the complete genome was 26.2% A, 24.2% G, 23.2% T, and 26.3% C. The complete genome sequence of the BEV-GX1901 isolate was submitted in GenBank with the accession number MN607030.

The complete genome length of BEV-GX1902 was 7405 nt, excluding the poly(A) tail. A large ORF of 6525 nt, which encoded a 2175 aa long polyprotein precursor, was flanked by an 812 nt long 5′UTR and a 74 nt long 3′UTR. The P1, P2, and P3 regions were 2517 nt (839 aa), 1734 nt (578 aa), and 2265 nt (755 aa) long, respectively. The base composition of the complete genome was 25.5% A, 23.5% G, 23.8% T, and 25.1% C. The complete genome sequence of the BEV-GX1902 isolate was submitted in GenBank with the accession number MN607031.

### 3.3. Physicochemical Properties of the Virus

To determine the infectivity of BEV-GX1901 and BEV-GX1902, TCID_50_ was determined as previously described. Two days post-inoculation, the wells (cells) with cytopathic effects were counted. Results from three repeats showed that the TCID_50_ for BEV-GX1901 isolates was 10^−6.5^/0.1 mL, and for BEV-GX1902 it was 10^−6.34^/0.1 mL.

**Figure 1:**
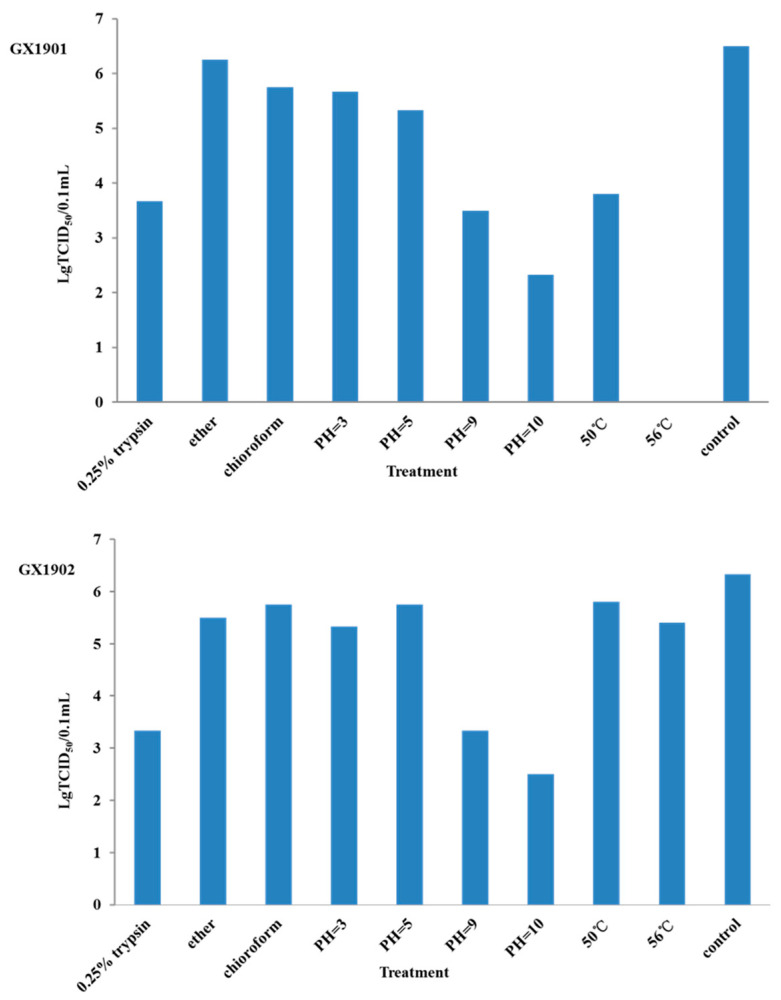
Physicochemical properties of BEV strains.

The infectivity of BEV-GX1902 was significantly reduced by the incubation at 50 °C and 56 °C for 1 h or the treatment with 0.25% trypsin. But the BEV-GX1901 isolates show more heat sensitivity after being treated under 50 °C for 1 h, and even completely lost its infectivity at 56 °C for 1 h, indicating that the virus of BEV-GX1901 is more sensitive to heat. The characterization of the physicochemical properties of two strains showed that they were resistant to the treatment in the low pH (3.0). The treatment of the two strains with chloroform/ether had no significant effects on its infectivity, suggesting it is a non-enveloped virus (Figure 1).

### 3.4. Phylogenetic Relationships and Evolutionary Analysis 

**Figure 2:**
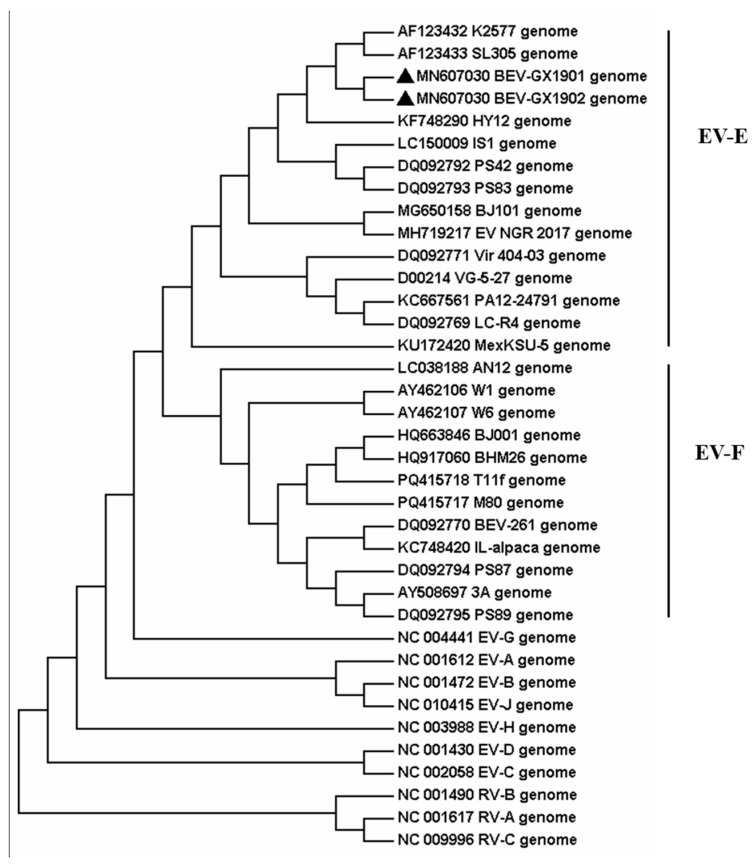
Phylogenetic analyses of the genome on the bovine enterovirus strains. Viruses are marked with symbols as follows: ▲ refers to the BEV strains obtained in this study.

**Figure 3:**
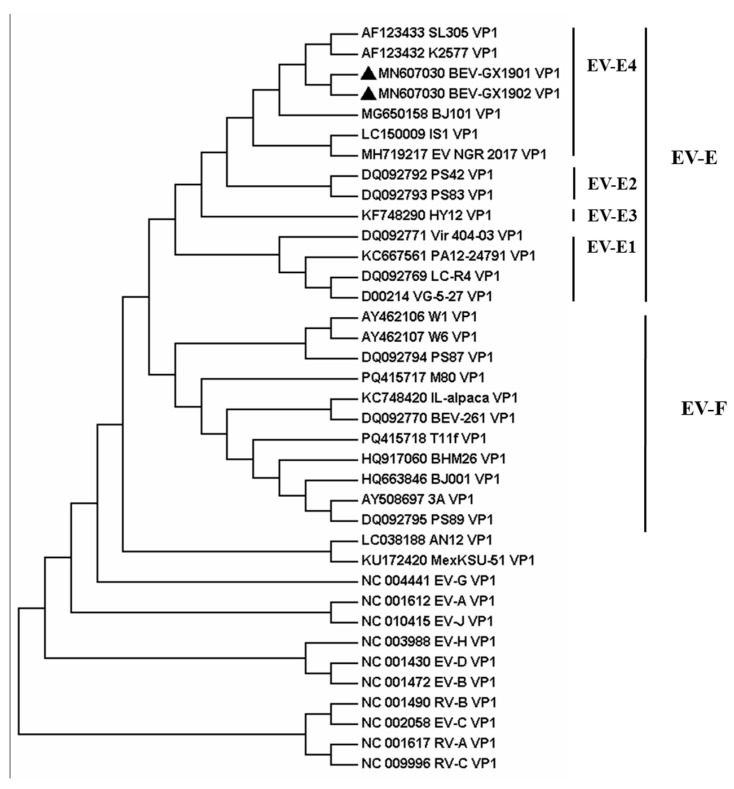
Phylogenetic analyses of VP1 on the bovine enterovirus strains. Viruses are marked with symbols as follows: ▲ refers to the BEV strains obtained in this study.

Then the two strains, BEV-GX1901 and BEV-GX1902, were located by pairwise nucleotide sequences that identified a 93.3% similarity with each other. To obtain more details of the two strains, phylogenetic analyses were applied by using the nucleotide sequences of the full-length genome and AA sequences of VP1 among BEVs, which were either determined in this study or by using currently available data from GenBank, and the reference bovine enterovirus sequences are shown in Figure 2 and Figure 3.

The result indicates that these two BEVs isolated in this study were classified as BEV E. All reference BEV strains are divided into E or F but cannot be divided into subtypes according to the whole genome sequence. Using the VP1 protein, which is normally the gold-standard gene, BEV-GX1901 and BEV-GX1902 are divided into E4 subtypes and together with strains isolated from Japan (IS1 strain), Austria (K2577, SL305), Germany (PS42, PS8), Nigeria (NGR-2017), and North China (BJ101), are almost all published E4 strains with total genome data for now. BEV-GX1901 and BEV-GX1902 were most closely matched to the Japan strain and were more related to Austrian strains than other isolated strains, including the strains isolated from Beijing, China. The VP1 genes of these two isolates showed a 76.7–90.9% nt shared identity with other strains of BEV-E4.

**Figure 4:**
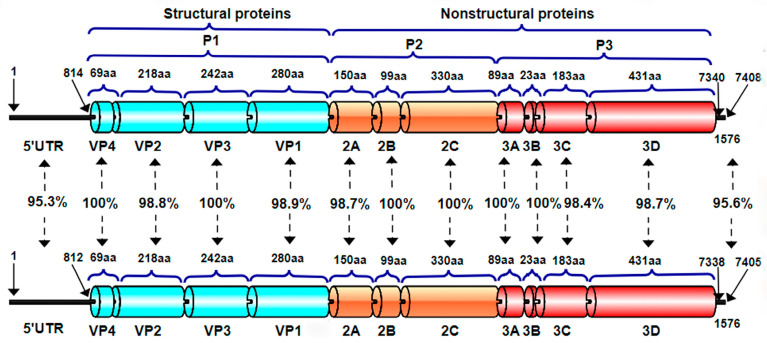
Nucleotide and amino acid identities between BEV-GX1901 and BEV-GX1902 by comparisons of subgenomic regions.

In the gene-by-gene or amino acid-by-amino acid comparison of the two strains (Figure 4), we found there are differences in 5′UTR and 3′UTR, and the identity was 95.3% and 95.6%, respectively. In structure proteins, there are differences in VP2 and VP1, and the identity was 98.8% and 98.9%, respectively. In unstructured protein, there are differences in 2A, 3C, and 3D, and the identity was 98.7%, 98.4%, and 98.7%, respectively.

## 4. Discussion

The pathogenicity of the BEV is somewhat controversial. Some people believe that BEV only causes mild diarrhea or a recessive infection in healthy cattle with weak pathogenicity, while others believe that BEV can cause respiratory diseases together with diarrhea, a decreased milk yield, orchitis, padermatitis, mucositis, abortion, stillbirth, cecal colitis, and other clinical symptoms [16]. This may be due to the fact that different serotypes of viruses have different propensities for various tissues to cause different disease forms. Furthermore, the pathogenicity mechanism is not very clear. Since its clinical symptoms are difficult to replicate in clinical experimental animal models, studies on the interaction between BEV and the host and its pathogenic mechanism are still in the preliminary stage.

So far, BEV has spread all over the world, with infection rates ranging from 17.6% to 80% [17,18,19]. The BEV infection is a new infectious disease occurring in China in recent years, which has caused serious harm to the healthy development of the cattle industry. The first Chinese BEV strain was isolated and identified from calves with diarrhea in Inner Mongolia, China, in 2011. Subsequently, in 2013, researchers isolated the BEV from diarrheal stool samples of lactating cows in Shandong Province, China. In the same year, Peng et al. isolated BEV from the fecal swabs of cattle suffering from severe diarrhea in Beijing, China, and named it BEV-BJ001, which was classified as BEV-F [20]. In 2014, Zhu et al. isolated and identified a novel enterovirus E isolate HY12 from cattle with severe respiratory and enteric diseases in Jilin, China [17]. In 2015, Zhang et al. isolated and identified an enterovirus E isolate HLJ-3531/2013 from fecal samples of lactating cows in Shanxi, China [7]. The above results indicated that BEV-E and BEV-F both appeared in China, and there were great differences between the two serotypes. At the same time, most of the isolates in China belonged to new strains, which caused certain difficulties in the investigation and diagnosis of the molecular epidemiology of this disease.

In this study, two strains of BEVs were isolated from the diarrheal feces of the same farm. It is well known that bovine diarrhea is caused by a variety of causes, mainly infectious diarrhea and non-infectious diarrhea. And the former is the main cause of morbidity and mortality in calves. Clinically, it is characterized by diarrhea and fevers, resulting in dehydration, an imbalance of the water and salt metabolism, acidosis, and even death. The pathogens that cause infectious diarrhea mainly include viruses, bacteria, and parasites. Among them, the viral pathogens mainly include BCV, BoAstV, BKV, BRV, and BVDV. On the basis of clinical signs, it was suspected that BCV, BoAstV, BKV, BRV, or BVDV were involved, but only BEV was detected from this sample, indicating the role and importance of a laboratory diagnosis in confirming the etiology. Subsequently, fecal samples were processed to inoculate the MDBK cells to be isolated and purified. Finally, we isolated two strains capable of stably producing CPEs, named BEV-GX1901 and BEV-GX1902, respectively. The complete genome sequences of BEV-GX1901 and BEV-GX1902 isolates were submitted in GenBank with accession numbers MN607030 and MN607031, respectively.

To better understand the differences between BEV-GX1901 and BEV-GX1902, the time of the cytopathic effect (CPE) production, TCID_50_ measurement, trypsin sensitivity test, and heat resistance test were studied. After incubating with the inoculum of BEV-GX1901, MDBK cells showed a typical CPE as early as 24 h. However, the typical CPE of BEV-GX1902 was produced at 48 h. This indicates that BEV-GX1901 is more virulent. Results show that the TCID_50_ for BEV-GX1901 and BEV-GX1902 isolates is 1 × 10^−6.5^/0.1 mL and 1 × 10^−6.34^/0.1 mL, respectively. And they both had high virulence titers, but the BEV-GX1901 strain’s virulence titer was higher than the BEV-GX1901 strain’s. To further characterize these two BEVs, the virulence titer of BEV-GX1901 has dropped down after treating it with the 0.25% trypsin and heat treatment at 50 °C for 1 h, but the TCID_50_ for BEV-GX1902 isolates has no significant change. And the BEV-GX1901 isolate completely lost infectivity at 56 °C for 1 h, indicating it is sensitive to the heat treatment at 56 °C. However, BEV-GX1902 also had a certain virulence titer. These physical and chemical properties determine that the virus can survive for several months in the natural environment. It also shows that BEV is very stable in the environment, and, thus, it has been proposed that this virus could be a useful indicator of water and environmental contamination [8,21].

Phylogenetic trees are constructed from the whole genomes of the isolates BEV-GX1901 and BEV-GX1902 and the reference strains. It is found that the two strains belonged to the same branch as the Australian strains K2577 and SL305 and the Japanese strain IS1, and they both belonged to the BEV-E type. The sequence of the VP1 gene provides an important source of information for classifying enteroviruses and differentiating between the serotypes [9,10,22]. A nucleotide identity of <75% and an amino acid identity of <88% were used as criteria for demarcating a new serotype [11]. Our isolates fulfill these criteria, with 91.4–96.4% amino acid and 76.7–90.9% nucleotide mean identities in the VP1 region, which closely matched the BEV-E4 Australian strains K2577 and SL305 and the Japanese strain IS1. And the two viruses isolated in this study are classified into the EV-E4 species based on these criteria.

According to the above analysis, the two isolates are closely related to the Australian strains, K2577 and SL305, and the Japanese strain IS1. And these two strains of the BEV were isolated from the feces of cattle imported from Australia, suggesting that the spread of BEV may be related to cattle trade and transportation. In recent years, with the strong support of the government, Guangxi has vigorously developed the cattle breeding industry. However, the farm needs to import cattle from outside the province to expand into a large-scale farm due to the few local cattle sources in Guangxi. Therefore, it is necessary to pay attention to the quarantine of diseases in cattle to avoid the introduction of disease.

In addition, this study also compared the 5′UTR and 3′UTR nucleotide sequences and ORF amino acid sequences of BEV-GX1901 and BEV-GX1902. The nucleotide sequences of 5′UTR and 3′UTR of these two strains were found to be more different, with a 95.3% and 95.6% homology, respectively. This was followed by the VP2, VP1, 2A, 3C, and 3D of the two strains, with an amino acid homology ranging from 98.4% to 98.9%. It is speculated that these loci/amino acid variants lead to their differences in physicochemical properties.

Recombination has an impact on the evolution, virulence, and transmissibility of many RNA viruses, including EVs [15,23,24]. It is generally believed that the molecular mechanism of recombination is the template conversion by RNA polymerase through the intermediate step of the replication fragment or the independent replication association of RNA molecules [25,26]. Recombination requires two different viruses to infect cells at the same time. Therefore, the recombinant strains need to have the same tissue tropism. Moreover, recombination often occurs between genomes with a large nucleotide similarity. As for EVs, recombination often occurs between different serotypes within the same EV group [27]. Gene recombination is common in the enterovirus. It is found that some secondary structures in the genome can promote inter-type recombination and intra-type recombination [28,29,30]. Recombination usually eliminates the effects of cumulative mutations. In addition, recombination may also recombine genes with different dominant properties into a new genome, resulting in drug resistance, immune evasion, virulence evolution, and so on. For example, recombination plays an important role in the development and enhancement of virulence in the vaccine-derived poliovirus. When the Sabin attenuated vaccine strain is recombined with other EV epidemic strains, the new recombinant strain may have a strong neurovirulence and transmission ability, which may easily cause new outbreaks [31,32]. Similarly, recombination events involving BEV have also been reported [4,33]. However, we did not find any evidence of recombination between these two strains. Therefore, further studies on these two strains should be conducted.

However, the pathogenicity of BEV is still unknown. There is a possibility that all viruses can cause diarrhea. To clarify the determinants of the pathogenicity of BEVs, an experimental infection based on a reverse genetic analysis is necessary.

## 5. Conclusions

In this study, two strains of BEV-E4 from the feces of newly transported cattle with diarrhea were isolated and characterized by purification, physicochemical characterization, and a viral genomic comparative analysis. In the comparative analysis, these two strains were more closely related to Australian strains K2577 and SL305 and the Japanese strain IS1, which may be related to the recent introduction from Australia and other places. And their effects on cattle diseases need to be further studied.

## Data Availability

The datasets supporting the conclusions of this article are included within the article. Accession numbers for the isolated genome sequences data are MN607030-MN607031 and have been deposited in the NCBI Genbank.
